# R18C is a new viable P2-like bacteriophage of rabbit origin infecting *Citrobacter rodentium* and *Shigella sonnei* strains

**DOI:** 10.1007/s00705-019-04424-5

**Published:** 2019-10-23

**Authors:** Domonkos Sváb, Balázs Horváth, Manfred Rohde, Gergely Maróti, István Tóth

**Affiliations:** 1 Institute for Veterinary Medical Research, Centre for Agricultural Research, Hungarian Academy of Sciences, Budapest, Hungary; 2 Seqomics Biotechnology Ltd., Mórahalom, Hungary; 3 Central Facility for Microscopy, Helmholtz Centre for Infection Research, HZI, Brunswick, Germany; 4 Institute of Biochemistry, Biological Research Centre, Hungarian Academy of Sciences, Szeged, Hungary

## Abstract

Here, we report a novel virulent P2-like bacteriophage, R18C, isolated from rabbit faeces, which, in addition to *Escherichia coli* K-12 strains, was able to be propagated on *Citrobacter rodentium* strain ICC169 and a range of *Shigella sonnei* strains with high efficiency of plating (EOP). It represents the first lytic bacteriophage originating from rabbit and the first infectious P2-like phage of animal origin. In the three characteristic moron-containing regions of P2-like phages, R18C contains genes with unknown function that have so far only been found in cryptic P2-like prophages.

**Electronic supplementary material:**

The online version of this article (10.1007/s00705-019-04424-5) contains supplementary material, which is available to authorized users.

P2-like bacteriophages are a group of phages that infect γ-proteobacteria and belong to the family *Myoviridae* (reviewed by Christie and Calendar [1]). They are frequently found as cryptic prophages within the genome of several strains of various species in the family *Enterobacteriaceae*, including pathogenic and non-pathogenic *Escherichia coli,* various serovars of *Salmonella enterica,* and strains of *Klebsiella pneumoniae*, *Yersinia spp.* and *Erwinia carotovora* [2]. P2-like phages, also called P2-related phages, are classified as members of the subfamily *Peduovirinae*, which is further divided into two subgroups: HP1-like and P2-like viruses [1]. At the genome level, members of the latter subgroup are generally viewed as rather conserved [3], and the functions of their genes are relatively well studied [1].

Here, we report the characterization of bacteriophage R18C isolated from rabbit faeces (kindly provided by László Makrai, University of Veterinary Medicine, Budapest, Hungary), which proved to be a new member of viable P2-like viruses.

The cell-free supernatant of the culture was spotted on layered soft agar plates containing *E. coli* K-12 strain MG1655 [4]. A single characteristic plaque was picked using a sterile toothpick and propagated subsequently on the same strain. The host spectrum and efficiency of plating (EOP) were also assessed by spot assay on layered soft agar plates containing the respective strains according to standard protocols described in detail by Tóth et al. [5]. It was found that besides lysing *E. coli* K-12 (the base titer was 2 × 10^5^ plaque-forming units per milliliter (PFU/ml)), phage R18C was propagated with an 10^4^ times higher efficiency of plating (EOP) on *Citrobacter rodentium* strain ICC169 [6], as well as on two *Shigella sonnei* strains. *S. sonnei* 75/02 is a recently characterized Shiga-toxin-producing strain [7], and R18C lysed it with the same EOP as it did strain ICC169, while on *S. sonnei* 866-F [8] the EOP was 3×10^3^. Enteroinvasive *E. coli* (EIEC) strain 548 of the O124 serogroup [9] also proved to be susceptible, with an EOP of 0.2.

Morphological investigation was performed by electron microscopy as described earlier [10]. The morphology of R18C was that of a myovirus, with an icosahedral head of 59 ± 2 nm in width and 61 ± 2 nm in length and a 157 ± 3 nm-long contractile tail (Fig. 1).

**Fig. 1:**
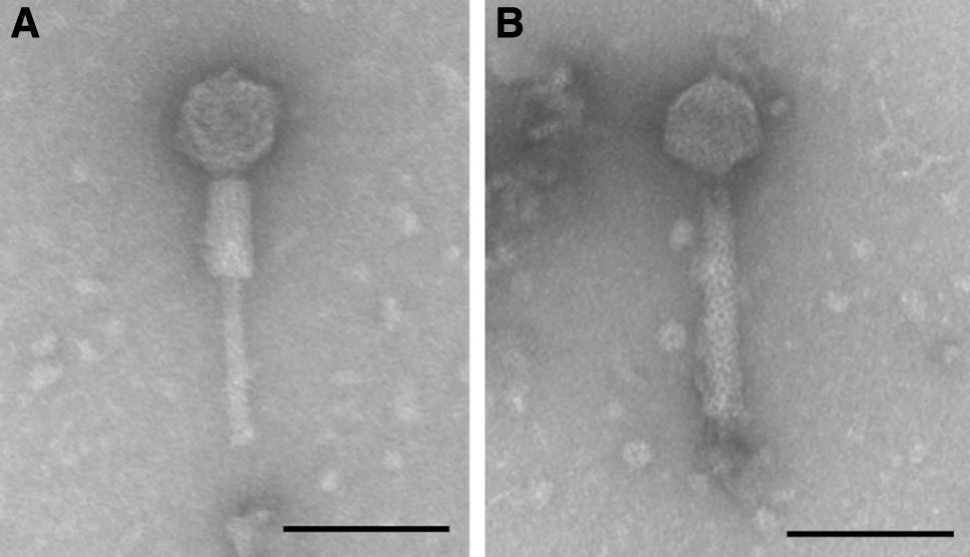
Transmission electron micrograph of bacteriophage R18C showing *Myoviridae* morphology with a contracted (A) and an uncontracted tail (B). Size measurements were performed on 15 intact individual phages. The bar represents 100 nm

A one-step growth experiment was performed in three parallel runs on *C. rodentium* ICC169 according to the protocol described by Bassiri [11]. The latent period of R18C was found to be 30 minutes, and the burst size was 100 new phage particles per cell on this host strain (Supplementary Fig. 1).

Phage DNA was isolated by the phenol-chloroform method [12] with the modifications described by Tóth et al. [5]. Genomic DNA sequencing libraries were prepared using a Nextera XT kit (Illumina, Eindhoven, NL). Sequencing was performed using a NextSeq/MiSeq Mid-Output Reagent Kit v2 (2 × 150 bp) on an Illumina NextSeq 500 / MiSeq sequencer. The average read length was 243 nucleotides, with 81x average coverage. Sequence assembly was performed using Spades 3.13.0 [13]. The genome sequence was annotated using the RAST server [14]. Homology searches for nucleotide and protein sequences were performed using the tools available on the NCBI website. Whole-genome comparisons were made with Easyfig [15], and phylogenetic analysis was performed with VICTOR for whole genomes, using the *d*_0_ distance formula [16]. The genome of bacteriophage R18C is a linear double-stranded DNA with a length of 31,834 nt and a GC content of 51.6%. This small genome size indicates that R18C is a dwarf myovirus [17]. The genome contains 45 protein-coding CDSs, a. comprehensive list of which is shown in Supplementary Table 1. The genome sequence was deposited in the GenBank database under the accession no. MN016939.

Homology searches and comparisons showed that R18C is a close relative of the P2-like bacteriophages, and its genome organization and order of ORFs with known function strictly corresponds to those of the prototype phage P2 and other viable phages of the P2-like viruses group. The end positions of the genome were also determined based on homologies, and they were later confirmed by the electrophoretic pattern of the genomic DNA after digestion with *Pst*I restriction endonuclease as well (Supplementary Fig. 2).

Phylogenetic analysis of the whole genome (Supplementary Fig. 3) indicated that the closest lytic relative of phage R18C within the group is bacteriophage WPhi [18] (GenBank no. AY135739.1; 83% coverage and 97.69% identity). However, the whole-genome phylogeny also showed that there are several prophages in *E. coli*, and even *E. albertii*, that are more closely related to R18C than either of the viable P2-like phages (Supplementary Fig. 4).

In P2-like phage genomes there are three regions that are known to carry morons, genes that are unnecessary for the life cycle of the phage (reviewed by Christie and Calendar [1]). These three regions in phage R18C showed markedly different genetic content when compared to other infectious P2-like viruses (Fig. 2).

**Fig. 2:**
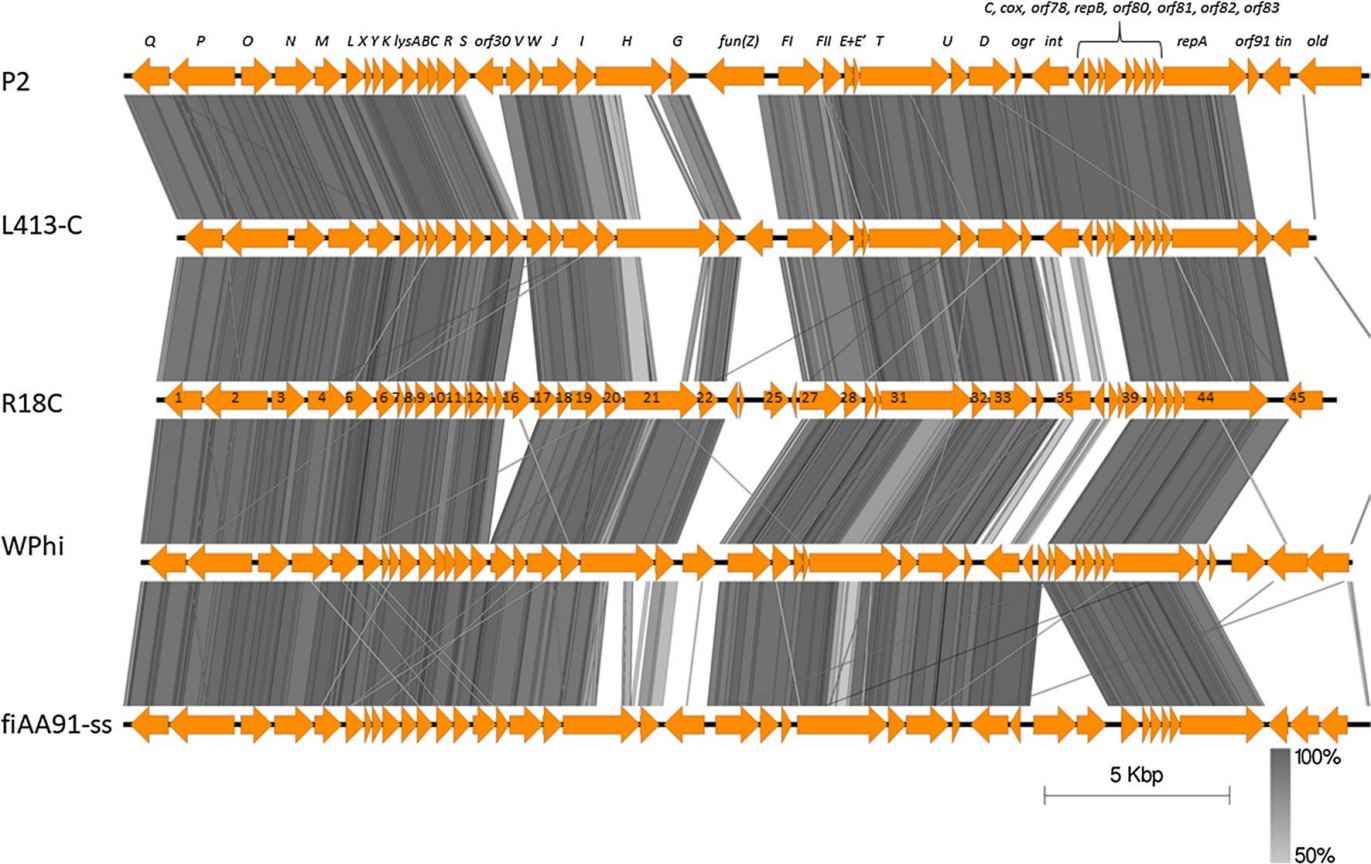
Alignment of lytic P2-like bacteriophage genomes made with EasyFig using the BLASTN algorithm. Grey lines connect regions with nucleotide identity > 50%, the darker color corresponds to a higher percentage of identity. The original designations of the ORFs of the P2 phage are shown at the top. ORF numbers are written in the arrows representing the R18C ORFs

The first such region is the TO region, between the genes encoding the replication protein A and capsid packaging protein Q (ORFs 44 and 1), named after the *tin* and *old* genes, which occupy this position in the prototype P2 phage and encode phage immunity genes against T-even and lambdoid phages, respectively. It is noted for carrying the gene cluster encoding the cytolethal distending toxin (CDT) type V in multiple P2-like prophages, which are harbored by *E. coli* strains of the O157 serogroup [8, 19]. In the case of phage R18C, the region falls to the 3’ end of the phage sequence and contains ORF45, which is annotated as hypothetical protein (position 30,367-31,452). Its closest homologues with 100% coverage are found in seven sequenced *Salmonella* strains, in one of which (GenBank no. CP032816.1) it is annotated as a pseudogene termed ‘cell filamentation protein Fic.’

Instead of the short, 261-bp-long ORF30 of the original P2 phage, there is a 741-bp-long ORF annotated as a hypothetical protein between baseplate assembly protein V and the phage tail completion protein S (ORF16, position 9,380-10,120, Fig. 2). A BLAST search of the GenBank database gives only seven highly similar hits, all of them in prophages within genomes of *E. coli* strains, in all cases annotated as ‘hypothetical protein.’

In the location of the fun(Z) phage immunity-protein-encoding gene, two short ORFs (no. 23 and 24, 219 and 279 bp long) annotated as encoding hypothetical proteins were found (position 15,385-15,853), flanked by relatively long non-coding regions. This location currently has 15 homologues in GenBank. In the cases where there is an annotation available, ORFs with the same length and similar annotations are found, with ORF24 annotated as ‘DUF3606-domain-containing protein’ in some cases.

In the case of all these moron ORFs, a protein motif search with PSI-BLAST did not yield any additional information regarding their possible structure or function.

In summary, we isolated and characterized bacteriophage R18C from rabbit faeces, which is able to lyse *C. rodentium* and *Shigella sonnei* with a high EOP. Phage R18C is a member of the P2-like viruses within the subfamily *Peduovirinae*, carrying a set of rare morons with unknown functions in its insertion sites. To our knowledge, R18C is the first infectious bacteriophage isolated from rabbit, and the first P2-like phage of animal origin. It is only the fifth viable phage characterized from this group, and the first P2-like phage described that is capable of lysing *C. rodentium,* and to our knowledge, R18C is the first P2-like phage that has had its burst size measured. As *C. rodentium* is frequently used as a model organism for the pathogenesis of enteropathogenic and enterohemorrhagic *E. coli* (EPEC and EHEC, [20]), a new bacteriophage capable of infecting it could be a valuable asset in future modeling experiments, and it could help in better understanding the dynamics of phage-host interactions as well. Determining the possible function of the morons it contains, as well as more detailed analysis of its genes related to host specificity, could reveal the role of this phage in the evolution and ecology of its hosts.

## Electronic supplementary material

Below is the link to the electronic supplementary material.

**Supplementary Table 1** List of ORFs identified in the genome of bacteriophage R18C (GenBank accession no. MN016939), their closest homologues, and their assigned functions (XLSX 13 kb)

**Supplementary Figure 1** One-step growth curve of bacteriophage R18C on *Citrobacter rodentium* strain ICC169. The initial number of phage particles was 2×10^6^, and samples were collected every 10 minutes. The PFU value is given for the whole volume of the culture, which was 50 ml. (TIFF 1536 kb)

**Supplementary Figure 2** Agarose gel electrophoresis of *Pst*I digested R18C phage DNA (A) and *Hind*III*-*digested lambda phage DNA (B). Electrophoresis was performed in a 1.7% agarose gel with a constant current of 4 V/cm for 2.5 hours. Arrows point to the 3.5- and 4.5-kb fragments of the R18C genome, which resulted from *Pst*I cutting sites at positions 4574-4575 and 27764-27765, which are closest to the 5’ and 3’ end of the genome, respectively. (TIFF 411 kb)

**Supplementary Fig. 3** Phylogeny of lytic P2-like bacteriophages based on the whole-genome sequence, made with VICTOR. GenBank accession numbers: P2, AF063097.1, WPhi: AY135739.1; L-413C, AY251033.1; fiAA91-ss, KF322032.1; R18C, MN016939 (TIFF 1093 kb)

**Supplementary Fig. 4** Whole-genome-based phylogeny of P2-like bacteriophages and prophages made with VICTOR. Ten of the first prophages among the whole-genome-based BLAST hits with the highest BLAST score to R18C were chosen and included in the tree. GenBank accession numbers of the prophages are given in the figure, and those of the lytic phages are as follows: P2, AF063097.1; WPhi, AY135739.1; L-413C, AY251033.1; fiAA91-ss, KF322032.1; R18C, MN016939. (TIFF 2068 kb)
