# Supplementary figures and images for: R18C is a new viable P2-like bacteriophage of rabbit origin infecting Citrobacter rodentium and Shigella sonnei strains
Source: Arch Virol. 2019 Oct 23;164(12):3157–60. doi: 10.1007/s00705-019-04424-5 (PMC6823313; doi:10.1007/s00705-019-04424-5)

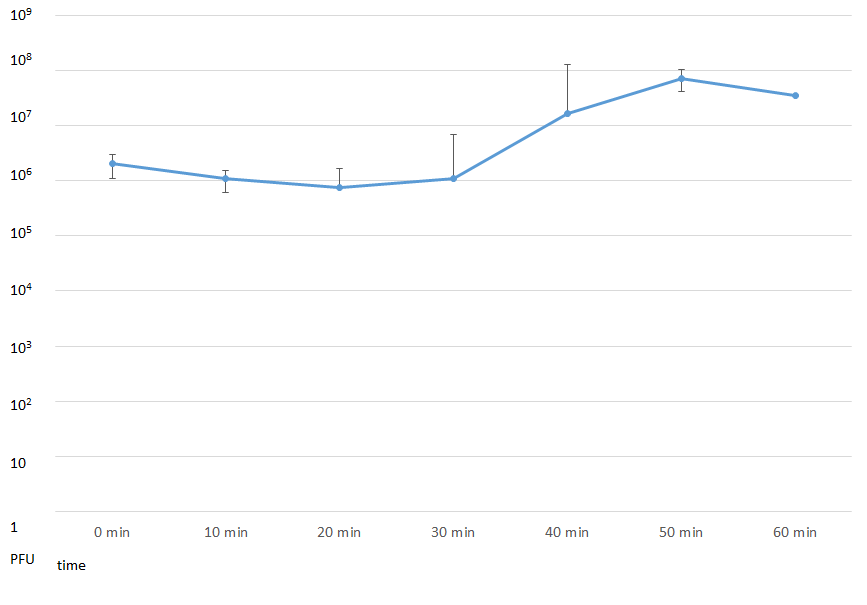

Supplement: Supplementary file 2 — Supplementary Figure 1 One-step growth curve of bacteriophage R18C on Citrobacter rodentium strain ICC169. The initial number of phage particles was 2×10^6^, and samples were collected every 10 minutes. The PFU value is given for the whole volume of the culture, which was 50 ml. (TIFF 1536 kb) [file 705_2019_4424_MOESM2_ESM.tif]

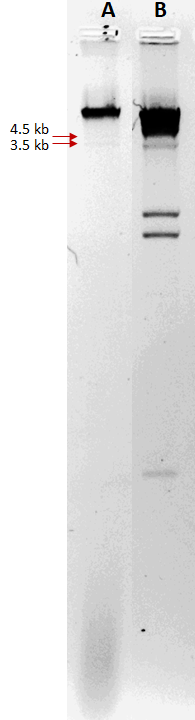

Supplement: Supplementary file 3 — Supplementary Figure 2 Agarose gel electrophoresis of PstI digested R18C phage DNA (A) and HindIII-digested lambda phage DNA (B). Electrophoresis was performed in a 1.7% agarose gel with a constant current of 4 V/cm for 2.5 hours. Arrows point to the 3.5- and 4.5-kb fragments of the R18C genome, which resulted from PstI cutting sites at positions 4574-4575 and 27764-27765, which are closest to the 5’ and 3’ end of the genome, respectively. (TIFF 411 kb) [file 705_2019_4424_MOESM3_ESM.tif]

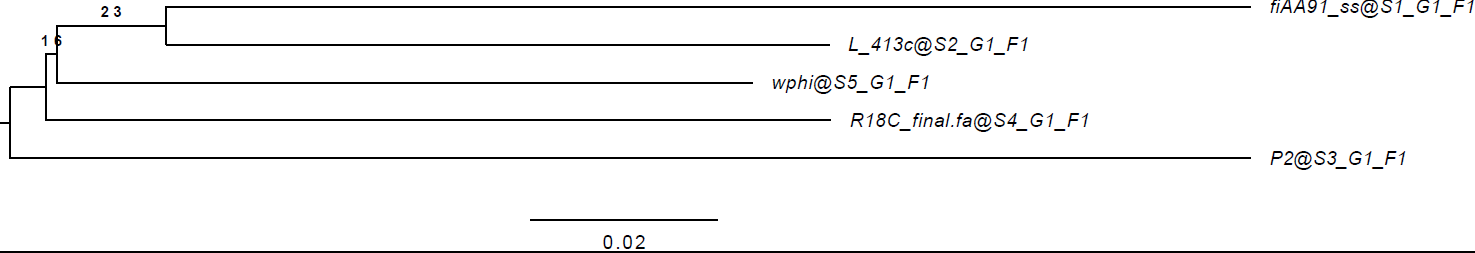

Supplement: Supplementary file 4 — Supplementary Fig. 3 Phylogeny of lytic P2-like bacteriophages based on the whole-genome sequence, made with VICTOR. GenBank accession numbers: P2, AF063097.1, WPhi: AY135739.1; L-413C, AY251033.1; fiAA91-ss, KF322032.1; R18C, MN016939 (TIFF 1093 kb) [file 705_2019_4424_MOESM4_ESM.tif]

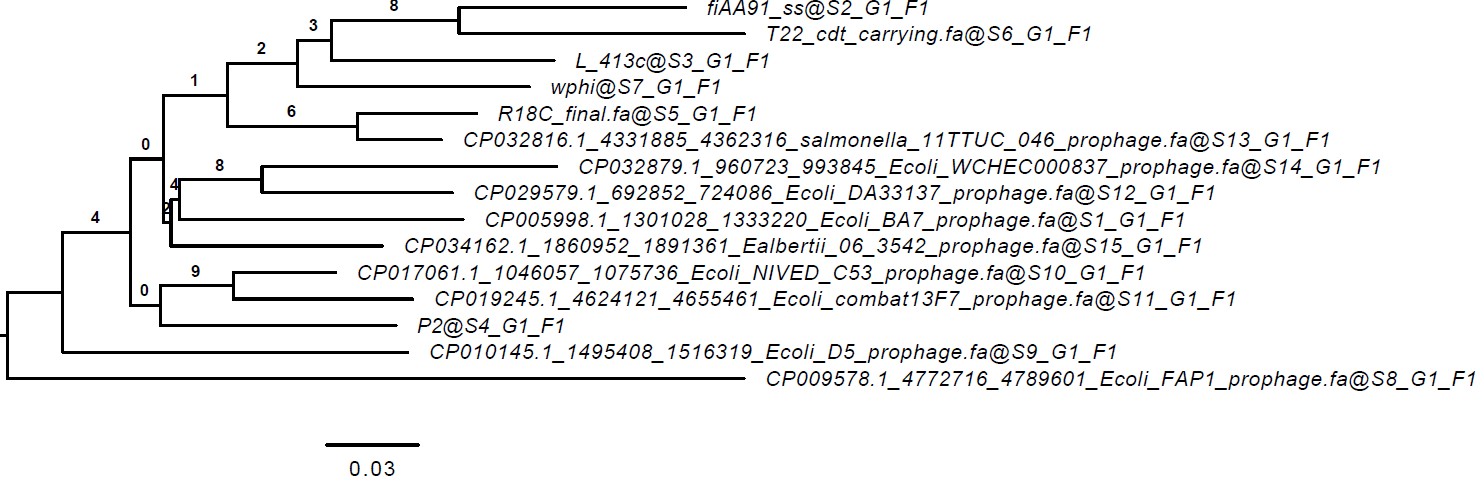

Supplement: Supplementary file 5 — Supplementary Fig. 4 Whole-genome-based phylogeny of P2-like bacteriophages and prophages made with VICTOR. Ten of the first prophages among the whole-genome-based BLAST hits with the highest BLAST score to R18C were chosen and included in the tree. GenBank accession numbers of the prophages are given in the figure, and those of the lytic phages are as follows: P2, AF063097.1; WPhi, AY135739.1; L-413C, AY251033.1; fiAA91-ss, KF322032.1; R18C, MN016939. (TIFF 2068 kb) [file 705_2019_4424_MOESM5_ESM.tif]
